# An implementation strategy package (video education, HIV self-testing, and co-location) improves PrEP implementation for pregnant women in antenatal care clinics in western Kenya

**DOI:** 10.3389/frph.2023.1205503

**Published:** 2023-11-17

**Authors:** Joseph Sila, Anjuli D. Wagner, Felix Abuna, Julia C. Dettinger, Ben Odhiambo, Nancy Ngumbau, George Oketch, Enock Sifuna, Laurén Gómez, Sarah Hicks, Grace John-Stewart, John Kinuthia

**Affiliations:** ^1^ Research & Programs, Kenyatta National Hospital, Nairobi, Kenya; ^2^ Department of Global Health, University of Washington, Seattle, WA, United States; ^3^ Department of Epidemiology, University of Washington, Seattle, WA, United States; ^4^ Departments of Pediatrics & Medicine, University of Washington, Seattle, WA, United States

**Keywords:** pre-exposure prophylaxis (PrEP), pregnancy, postpartum, implementation science, video education, HIV self testing, integration

## Abstract

**Background:**

Pre-exposure prophylaxis (PrEP) is recommended by the World Health Organization and the Kenyan Ministry of Health for HIV prevention in pregnancy and postpartum for women at risk for HIV. Integration of PrEP into antenatal care is promising, but delivery gaps exist in the face of healthcare provider shortages in resource-limited settings.

**Methods:**

Between May and November 2021, we conducted a difference-in-differences study (3 months pre-intervention data collection and 3 months post-intervention data collection) analyzing four intervention facilities, where the strategies were implemented, and four comparison facilities, where no strategies were implemented. We tested a combination of three implementation strategies—video-based PrEP information in the waiting bay, HIV self-testing, and dispensing of PrEP in the antenatal care rooms—to improve PrEP delivery. We compared absolute changes in the proportion of antenatal attendees screened for PrEP (PrEP penetration), the proportion receiving all PrEP-specific steps in a visit (HIV testing, risk screening, and PrEP counseling) (PrEP fidelity), and client PrEP knowledge, client satisfaction, and waiting time and service time (*a priori* outcomes); *post hoc,* we compared the proportion offered PrEP (PrEP offer) and completing HIV testing. We measured provider perceptions of the acceptability and appropriateness of the implementation strategies.

**Results:**

We observed significant improvements in PrEP penetration, PrEP offer, satisfaction, and knowledge (*p* < 0.05) and improvements in fidelity that trended towards significance (*p* = 0.057). PrEP penetration increased 5 percentage points (*p* = 0.008), PrEP fidelity increased 8 percentage points (*p* = 0.057), and PrEP offer increased 4 percentage points (*p* = 0.003) in intervention vs. comparison facilities. Client PrEP knowledge increased by 1.7 out of 6 total points (*p* < 0.001) and client satisfaction increased by 0.7 out of 24 total points (*p* = 0.003) in intervention vs. comparison facilities. We observed no changes in service time (0.09-min decrease; *p* = 0.435) and a small increase in waiting time (0.33-min increase; *p* = 0.005). HIV testing among those eligible did not change (1.5 percentage point decrease, *p* = 0.800). Providers felt the implementation strategies were acceptable and appropriate (median acceptability: 20/20; median appropriateness: 19.5/20). However, absolute levels of each step of the PrEP cascade remained suboptimal.

**Conclusions:**

An implementation strategy package with video information, HIV self-testing, and co-location of medication dispensing enhanced PrEP delivery across several implementation outcomes and client satisfaction, while not substantially increasing wait time or decreasing provider-client contact time.

**Clinical trial registration:**

ClinicalTrials.gov , identifier, NCT04712994.

## Introduction

Pregnant and postpartum women in high HIV prevalence settings face an elevated risk of HIV acquisition due to biological and social factors (1, 2). Pre-exposure prophylaxis (PrEP) is a safe, effective, and acceptable intervention for use during pregnancy and postpartum (3–7). It is recommended by the World Health Organization and several countries’ guidelines (8). Despite this endorsement, the reach and coverage of PrEP during this period remain suboptimal globally due to a range of implementation challenges (9). A systematic review of implementation science studies of PrEP in pregnancy and postpartum noted that implementation challenges exist at the intrapersonal, interpersonal, community, and systems levels. However, most implementation strategies tested to improve implementation intervened at the intrapersonal and interpersonal levels, rather than focusing on the systems level. In addition to demand-generating activities, supply-side interventions to improve implementation are needed to fully realize the population-level benefits of PrEP for HIV prevention (10).

PrEP can be delivered in vertical siloed programs—such as through HIV care clinics (11, 12)—or horizontally in integrated programs—such as through maternal and child health (MCH) or family planning clinics (13–16). Pregnant and postpartum women report preferring integrated service delivery for reasons of convenience and reduced stigma (17). In order to provide integrated PrEP delivery, healthcare workers (HCWs) require specific training in PrEP counseling, prescribing, and documentation. HIV testing providers have a higher volume of clients to test each day in order to initiate PrEP, and clinics within a facility need to determine where to dispense PrEP (either from a central pharmacy or a clinic-specific setting).

Kenya was an early adopter of integrated PrEP delivery in pregnancy and postpartum; Kenyan research teams have conducted a series of qualitative investigations, implementation projects, and trials to identify optimal approaches for integrated PrEP delivery (16–24). An early implementation project focusing on developing integrated models for PrEP delivery in MCH clinics highlighted that services can be delivered by a single nurse delivering antenatal/postnatal and PrEP services or by multiple nurses sequentially, with the service delivery model selection depending on clinic organization, space, and staffing. PrEP activities take additional time, with a median of 13 min for PrEP education and counseling among clients who did not initiate PrEP and 18 min for clients who initiated PrEP; compared to average antenatal clinic (ANC) service times of 9 min and waiting time of 13 min, this represents substantial additional time spent by both clients and HCWs (23). HCWs highlighted insufficient staffing, insufficient staff PrEP knowledge, insufficient space, and high patient volumes as the most impactful barriers experienced while delivering PrEP to pregnant and postpartum populations across 55 facilities in Kenya (25). Implementation strategies to address these additional time demands and patient activity volumes associated with integrated PrEP offers could meaningfully increase the reach and coverage of PrEP for this priority population.

In this study, we tested a combination of three implementation strategies to decrease client waiting time, improve coverage of PrEP education and PrEP offer, improve PrEP knowledge, and maintain satisfaction for clients and HCWs. The package included video education, HIV self-testing (HIVST) for repeat HIV testing, and PrEP dispensing in MCH clinics.

## Methods

### Setting & design & population

This study is registered at ClinicalTrials.gov (NCT04712994). This study was conducted in three counties in western Kenya: Kisumu, Siaya, and Homa Bay counties. These counties have relatively high HIV prevalence. We focused on MCH clinics at each site, which provide ANC, postnatal (PNC), and child welfare services. We engaged eight facilities in a difference-in-differences design with 3 months of baseline data collection (May through July 2021) and 3 months of intervention period data collection (August through November 2021); four facilities were never exposed to the implementation strategy package and four facilities were exposed to the implementation strategy package during the second 3-month period. The distribution of study sites in each county was balanced between the intervention and comparison facilities (Supplementary Table S4). We aimed to select facilities that were of similar size and staffing to other facilities in the region to enhance external validity and generalizability. However, there were factors in the selection process that may have limited generalizability. The eight facilities were selected from among a list of facilities that had previously engaged in either a research trial (24), a demonstration project with staffing support (16), or a mentorship model with no staffing support (22). All prior work was finished at each site prior to engagement for our study and no additional staff were supporting PrEP delivery in MCH clinics. Facilities that were selected for these research or demonstration projects in the past tended to have somewhat better resourcing, including physical space availability (26). We further note that these eight clinics are not representative of smaller facilities in the region, which were systematically missing due to low numbers of clients served, limiting feasibility as research sites. The original study design was intended as a controlled interrupted time series, which employs the same pre-post and concurrent comparison clinic elements, but differs from the difference-in-differences design by controlling for linear temporal trends during each period; however, due to interruptions in data collection related to COVID-19, flooding, strikes, and other unanticipated events, the calendar time table was interrupted, necessitating the switch to a difference-in-differences analytic approach.

### Ethical approval

This study was reviewed and approved by the Kenyatta National Hospital/University of Nairobi Ethics & Research Committee (P907/11/2019) and the University of Washington Institutional Review Board (STUDY00008392). Facilities were engaged to participate by seeking the relevant county, sub-county, and site-level approval.

### Implementation strategy package

The implementation strategy package contained three components: (1) video education, (2) HIV self-testing (HIVST) for repeat HIV testing, and (3) PrEP dispensing in MCH clinics rather than in a central or HIV-specific pharmacy. The following descriptions focus on specification using the Proctor specification approach, highlighting actor, action, action target, temporality, dose, implementation outcomes targeted, and theoretical justification (27). Of note, this study did not employ any research staff or additional program staff to deliver clinical services and aimed to test strategies to improve implementation without additional human resources.

The video was created by a local videography company with prior experience creating engaging and informative videos for MCH audiences. The content of the informational video was developed by the study team and informed by quantitative and qualitative data from past PrEP in pregnancy studies (28). The story characters were developed to reflect the most common populations, including a married primigravida who did not know her partner's HIV status. The modes of PrEP information delivery featured in the video mirrored the methods reported to motivate women to initiate PrEP: PrEP-experienced peer conversations and HCW conversations. The PrEP-specific clinical information provided covered the required elements of PrEP counseling as outlined by the Kenyan National AIDS & STI Control Program. The video was presented in three languages (English, Dholuo, and Kiswahili) to reflect the common languages in the region and featured subtitles. The video employed a dramatized, soap opera style to mirror common popular TV programs; it was approximately 13 min in length [the average waiting time was 13 min for a similar population (23)]. The video was played in the waiting room of the MCH clinic at each intervention site on repeat; MCH clients could watch the video in a group setting in the waiting room. Clients were encouraged to ask questions about PrEP to the HCW they saw during their subsequent care. This strategy was selected in order to reduce the amount of time spent by HCW delivering standardized PrEP information, aiming to decrease waiting time and service time and increase PrEP penetration and fidelity. Video education has been used in numerous high-resource and some low-resource settings for HIV pre-test information provision and has shown to be either superior or equivalent to counselor-delivered information (29, 30).

HIVST is utilized in Kenya as a screening test and is endorsed in the national HIV testing services guidelines. The OraQuick test was procured through central government systems and provided by the site. Women were eligible to use the OraQuick for repeat HIV testing if they were not attending their first ANC visit; those attending the first ANC visit were required to complete standard HIV testing services. Privacy booths—such as those described by Oyaro et al. (31)—were provided near the waiting bays. Standardized pictorial and text instructions were provided as part of the OraQuick insert in both English and Kiswahili. Women collected their own samples, submerged them in the reaction fluid, and used a stopwatch to wait for the required 15-minute reaction time. Women read their own results and thereafter showed their test results to an HCW for confirmation of correct interpretation. Women whose HIVST was non-reactive did not undergo additional HIV testing and were considered eligible to initiate PrEP. Women whose HIVST was reactive or had any irregular result underwent standard HIV testing by the site HCW. This strategy was selected in order to reduce the amount of time spent by HCWs waiting for HIV test reactions to take place and to reduce the volume of clients needed to be served by the limited number of HIV testing providers at a given site.

For women who were offered PrEP and decided to initiate or continue PrEP, the pills were dispensed within the MCH clinic, rather than at a central or HIV-specific pharmacy, aligning with the strategy of co-location. This strategy was selected in order to eliminate additional waiting time and to reduce the potential stigma associated with receiving medication at the HIV-specific pharmacy.

### Implementation & service outcomes

We measured several PrEP implementation, service, and health outcomes (32), which are shown in Table 1. Our primary outcomes were PrEP penetration, PrEP fidelity, client satisfaction, healthcare worker acceptability and appropriateness (33), and waiting time and service time (*a priori* primary outcomes). PrEP uptake, PrEP continuation, PrEP adherence, and client PrEP knowledge were *a priori* secondary outcomes. PrEP offer and HIV testing completion were added as *post hoc* outcomes. During preparation activities for data collection, it was determined that it was not feasible to extract patient adherence information; this outcome was neither collected nor compared. Women were considered eligible for HIV testing in our analytic dataset if they had not had an HIV test within the past 6 months and were not known to be living with HIV prior to the visit. For proportions with conditional denominators (PrEP uptake, PrEP offer, and HIV testing), analyses were presented first for the conditional denominator (e.g., uptake among those offered PrEP) and second for the full denominator (e.g., uptake among all women seeking services). This approach was taken to show the relative and absolute changes. The percentages for each outcome within Table 1 were calculated directly from the observed data rather than the model-predicted levels, following the proportion definitions presented in Table 1.

**Table 1:** Difference in differences comparison of implementation, effectiveness, and service outcomes.

		Comparison sites				Intervention sites				Difference in difference [(Change in intervention sites)—(Change in comparison sites)] *Adjusted for first ANC*		
		Pre (*N* = 480)		Post (*N* = 478)		Pre (*N* = 480)		Post (*N* = 481)		Point estimate	Confidence interval	*p*-value
Outcome	Definition	*n* (%) or median IQR	*N*	*n* (%) or median IQR	*N*	*n* (%) or median IQR	*N*	*n* (%) or median IQR	*N*			
PrEP fidelity^b^	Proportion of women who receive all PrEP-specific steps in a HIV-testing visit, HIV-risk screening, PrEP-counseling/total women receiving antenatal or postnatal services [*Risk screening question: “Please answer yes if you were asked about any of the following behaviors today: (Kenyan risk assessment tool HIV risk factors)”]*	4 (2.5%)	161	5 (3.0%)	164	14 (8.3%)	168	28 (15.5%)	181	7.6%	(−0.2%, 15.4%)	0.057
HIV testing^d^	Proportion of women HIV tested among eligible candidates (not tested in past 6 months, not known HIV positive)	98 (60.1%)	163	107 (65.2%)	164	142 (84.0%)	169	144 (79.6%)	181	−1.5%	(−12.9%, 9.9%)	0.800
PrEP risk screening^d^	Proportion asked questions on HIV risk behavior characteristics (yes vs. no/don’t know)	43 (9.0%)	480	104 (21.8%)	478	146 (30.5%)	479	159 (33.1%)	481	−8.8%	(−15.9%, −1.8%)	0.013
PrEP penetration^b^	Proportion of women who are counseled about PrEP / total women receiving antenatal or postnatal services (“*Did anyone talk to you about PrEP today?*”)	17 (3.5%)	480	8 (1.7%)	478	30 (6.3%)	480	46 (9.6%)	481	5.4%	(1.4%, 9.3%)	0.008
PrEP offer^d^	Proportion offered to start or continue taking PrEP/total women receiving antenatal or postnatal services	8 (1.7%)	480	9 (1.9%)	478	6 (1.3%)	480	28 (5.8%)	481	4.4%	(1.5%, 7.2%)	0.002
	Proportion offered to start or continue taking PrEP (among those who were HIV negative and at high risk who were screened)	3 (15.8%)	19	6 (9.8%)	61	4 (5.1%)	78	13 (14.0%)	93	−5.9%	(−13.8%, 2.1%)	0.148
PrEP uptake^c^	Proportion initiated PrEP today (among those offered)	1 (12.0%)	8	1 (11.0%)	9	0 (0%)	6	0 (0%)	28	–	–	–
	Proportion initiated PrEP today (among the full population)	1 (0.2%)	480	1 (0.2%)	478	0 (0%)	480	0 (0%)	481	–	–	–
PrEP continuation^c^	Already taking PrEP and will continue to take PrEP (among those offered)	1 (12.5%)	8	2 (22.2%)	9	1 (16.7%)	6	1 (3.6%)	28	–	–	–
	Already taking PrEP and will continue to take PrEP (among the full population)	5 (1.0%)	480	10 (2.1%)	478	9 (1.9%)	480	6 (1.2%)	481	–	–	–
Service time^b^	Number of minutes receiving services from health care workers	16 (10–33)	192	14 (9–27.5)	192	18 (12–36)	192	17 (11–29)	192	−0.09^a^	(−0.33, 0.14)^a^	0.435^a^
Waiting time^b^	Number of minutes spent waiting to receive services	37 (14.5–74)	192	29 (14–46.5)	192	49 (22.5–77)	192	55 (31–80)	192	0.33^a^	(0.10, 0.56)^a^	0.005^a^
Client satisfaction^b^	Satisfaction on a scale of 0–24 points; 6 questions for clients to assess their satisfaction with services received at the facility; Likert scale (worst to best: 1–5)	23.0 (20.0–23.0)	480	23.0 (20.0–24.0)	478	21.0 (19.0 −23.0)	480	23.0 (21.0–23.0)	481	0.66	(0.22, 1.09)	0.003
HCW satisfaction^b^	Average on 4-item Intervention Appropriateness Measure (IAM) scale; Likert scale (disagree to agree: 1–5)	–		–		–		19.5 (16.0, 20.0)		–	–	–
	Average on 4-item Acceptability of Intervention Measures (AIM) scale; Likert scale (disagree to agree: 1–5)	–		–		–		20.0 (16.0, 20.0)		–	–	–
Client PrEP knowledge^c^	All correct answers (6 questions based on content covered in counseling sessions)	0 (0%)	480	5 (1%)	478	5 (1%)	480	56 (11.6%)	481	9.6%	(6.5, 12.8%)	<0.001
	Number of correct answers (0–6)	0.975	480	1.234	478	1.042	480	3.019	481	1.72	(1.45, 1.99)	<0.001

^a^ Not adjusted for visit type due to visit type not collected during time-and-motion activity.

^b^ Primary outcome.

^c^ Secondary outcome.

^d^ Post hoc outcome.

### Participant recruitment, enrollment, and data collection

Women seeking MCH services were approached after receipt of services between May and November 2021. Clients were eligible if aged ≥15 years and able to provide oral consent. Participants completed an exit survey with trained study nurses on a tablet using REDCap after all other regular care for their visit concluded. We assessed participant demographics, HIV risk screening and counseling, PrEP knowledge, and satisfaction with services offered that particular day. Separately, we used time and motion cards designed to collect “time in” and “time out” at different service delivery locations. The study nurse would conduct oral consent with the women at the MCH entrance, document the “time of arrival” on the card, and give the woman the card to carry along. HCWs at different service delivery stations could complete the two time points (time in and time out). At intervention clinics during the post-intervention period, we approached all HCWs offering services in MCH and invited them to provide oral consent and complete a REDCap survey either alone using a computer link or with study nurses using a tablet. HCWs were given 2 weeks to complete the survey; several (typically two) follow-up attempts were made by phone; those who did not complete the survey in 3 weeks were excluded.

#### Data abstraction

We abstracted data without patient identifiers from PrEP registers noting the number who initiated and the number who continued PrEP aggregated by day. As these registers collected data facility-wide, it was not possible to determine where individuals initiated PrEP (e.g., MCH or another clinic) or determine who was pregnant or postpartum or a woman.

#### Data analysis

We summarized descriptive data using proportions, medians, and interquartile ranges, as well as means and standard deviations. We log-transformed waiting time and service time for this analysis and presented geometric means in analytic tables. We did not transform knowledge or satisfaction scores, although they were positively skewed, as transformations did not produce more normal distributions. We assessed the change associated with the implementation package using a difference-in-differences analytic approach, using a multi-level mixed-effect regression model with a random effect for the site, a binary term for intervention vs. comparison group, a binary term for pre/post time period, and an interaction term between the two. We additionally controlled for differences in the proportion of women seeking first antenatal care services through inclusion as a covariate (primary analysis) and presentation of analyses stratified by visit type (secondary analysis). We estimated the change associated with the implementation package as the interaction term and considered a change statistically significant at alpha ≤0.05. We conducted a basic optimization analysis for the PrEP steps in order to estimate the idealized scenario of the maximum number of women who might be offered PrEP and accept PrEP if PrEP counseling, PrEP risk assessment, HIV testing, and offer were optimized, without changes to the proportion accepting PrEP. We multiplied the total number of women in our sample by the proportion who had any risk indication for PrEP, the proportion who were eligible for HIV testing that day, the proportion who would have tested HIV negative (based on this dataset), and the proportion who would have accepted PrEP (based on this dataset). This yielded the maximum number who would have likely initiated PrEP in the idealized scenario of perfect penetration, fidelity, and offer and the observed proportions of uptake.

#### Contextual factors and temporal changes

**Figure 1:**
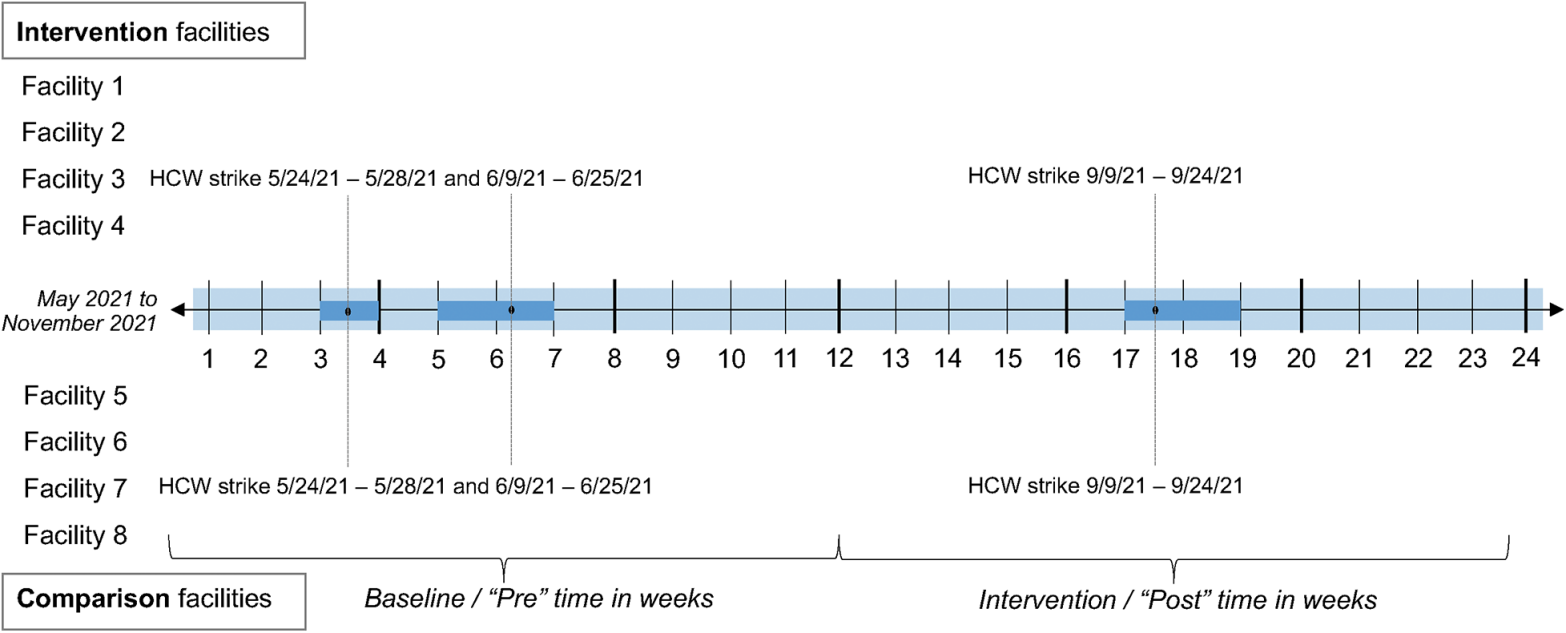
Timeline in weeks and background service delivery interruptions.

During the 3 months of pre-intervention and 3 months during the intervention, some events occurred either at or beyond facilities that may have impacted service delivery broadly or delivery of the implementation strategy package specifically. A timeline of these events and activities is shown in Figure 1. Because the frequency of interruptions was balanced between the intervention and comparison facilities, we did not conduct sensitivity analyses accounting for these interruptions.

## Results

### Demographic characteristics

We enrolled a total of 1,919 participants receiving MCH services during the 3 months pre-intervention and 3 months during the intervention [960 pre-intervention (480 in comparison and 480 in intervention sites) and 959 during the intervention (478 in comparison and 481 in intervention sites)]. Among women seeking MCH services, the median age was 25 [interquartile range (IQR): 22, 30] years, 21.5% were seeking first ANC visits, while 78.5% were seeking second or subsequent ANC visits or other MCH services. In comparing the demographic details between women at intervention vs. comparison sites in the pre-intervention vs. during intervention periods, we noted no differences in age and only slight differences in the proportion seeking a first ANC visit vs. other services (comparison sites: pre-intervention, 19.0%; post-intervention, 18.6%. Intervention sites: pre-intervention, 26.9%; post-intervention, 21.4%) (Supplementary Table S2).

### Baseline period

During the baseline period, PrEP penetration, PrEP fidelity, PrEP offer, and PrEP knowledge were low in both intervention and comparison clinics; there was substantial heterogeneity between sites in implementation outcomes (Supplementary Table S3). PrEP penetration ranged from 0%–10%, PrEP fidelity from 0%–16%, PrEP offer among eligible women from 0%–13%, and full complete PrEP knowledge from 0%–1.7%. In contrast, HIV testing was higher (ranging from 42%–95%), and satisfaction with services was high (ranging from 21 to 23 out of 24 points). Time spent waiting and receiving services ranged from 10.5–79 min and 12–25.5 min, respectively. As each clinic served as its own baseline measurement and comparison, we did not test for differences between intervention and comparison clinics in baseline implementation outcomes.

### Changes associated with the implementation strategy bundle

We used difference-in-differences analysis to assess the changes associated with the implementation strategy bundle. For our primary outcomes, the implementation strategy bundle was associated with significant increases in PrEP penetration, client satisfaction, and client PrEP knowledge and was associated with a significant but small magnitude increase in waiting time and no change in service delivery time. The implementation strategy bundle was also associated with a substantial improvement in PrEP fidelity but was only trending toward significance.

**Figure 2:**
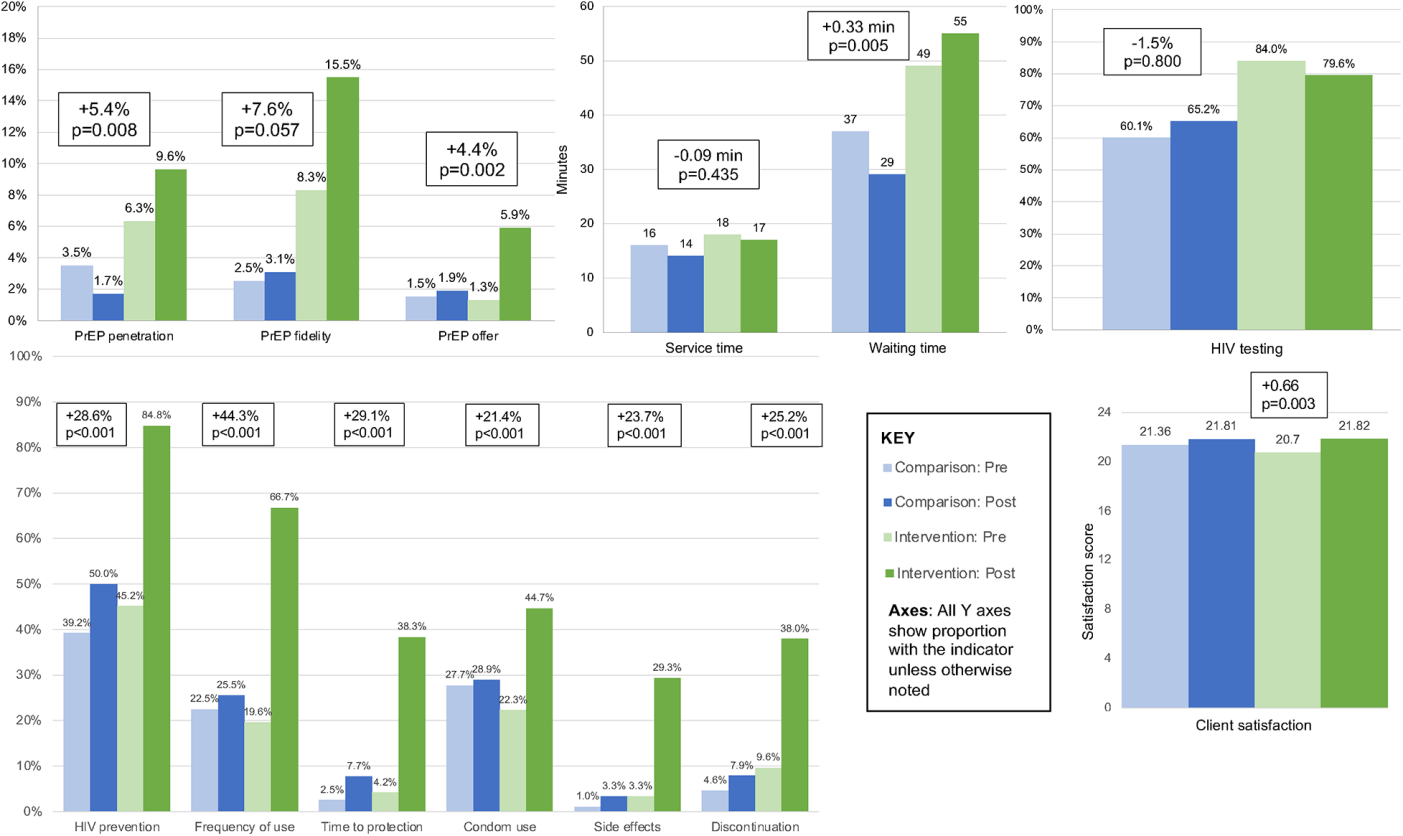
Difference in differences comparison of implementation, effectiveness, and service outcomes. Statistical test compares the difference between pre and post levels in the comparison to the intervention sites.

The implementation strategy bundle was associated with a PrEP penetration increase of 5.4% percentage points (95% CI: 1.4, 9.3%; *p* = 0.008) in intervention vs. comparison sites and reached a high of 9.6% in intervention sites (Table 1). The change in penetration was more pronounced among clients seeking first ANC services vs. any other visit type (12.7% vs. 3.4% percentage point increase, respectively) (Supplementary Table S1). PrEP fidelity increased by 7.6% percentage points (95% CI: −0.2%, 15.4%; *p* = 0.057) more in the intervention vs. comparison sites, reaching a high of 15.5%, but only trended towards significance (Table 1). The change in fidelity was more pronounced among clients seeking first ANC services vs. any other visit type (12.5% vs. 3.5% percentage point increase, respectively) (Supplementary Table S1). Despite the increase in PrEP fidelity, there was a significant and substantial decrease in the coverage of PrEP risk screening assessment [8.8% percentage point decrease (95% CI: −15.9%, −1.8%); *p* = 0.013] between intervention and comparison sites, reaching a high of 33.1%. While both the intervention and comparison sites increased in screening assessment, the increase was larger in the comparison sites, where two comparison sites had newly added screening desks midway through the test; this difference was comparable between clients seeking first ANC services and those with any other visit type (Table 1, Figure 2, Supplementary Table S1).

The implementation strategy bundle was associated with a PrEP knowledge increase of 9.6% percentage points (95% CI: 6.5, 12.8%; *p* < 0.001) in the intervention vs. comparison sites, reaching a high of 11.6%. This corresponded to an increase in 1.72 additional questions correct out of 6 total questions. Client satisfaction increased by 0.66 points (95% CI: 0.22, 1.09; *p* = 0.003) in the intervention vs. comparison sites, reaching a high of 23.0 out of 24 points. Client waiting time increased by 0.33 min (95% CI: 0.10, 0.56; *p* = 0.005) in the intervention vs. comparison sites, reaching a median of 55 min during the post-intervention period. Neither the changes in knowledge nor satisfaction were different between clients seeking first ANC services and those with any other visit type (Supplementary Table S1). Client service time did not substantially or significantly change [0.09-min decrease; (95% CI: −0.33, 0.14); *p* = 0.435]. Waiting and service times were not adjusted for visit type (Table 1; Figure 2).

In our secondary analyses, the implementation strategy bundle was associated with substantial and significant improvements in PrEP offer but not in HIV testing. HIV testing did not change significantly [1.5% decrease (95% CI: −12.9, 9.9%), *p* = 0.800], reaching a high of 84% in the baseline period of the intervention sites; HIV testing increased modestly in the comparison sites and decreased by a similar magnitude in the intervention sites. The difference was comparable between clients seeking first ANC services and those with any other visit type (Supplementary Table S1). PrEP offers among all women increased by 4.4% percentage points (95% CI: 1.5, 7.2%, *p* = 0.002), reaching a high of 5.8% among all women receiving services. The change in PrEP offer was more pronounced among clients seeking first ANC services vs. any other visit type (7.9% vs. 3.3% percentage point increase, respectively) (Supplementary Table S1). Among the subset of women who were HIV negative and had any high-risk factor, PrEP offer was somewhat substantially but not significantly lower [5.9% increase (95% CI: −13.8, 2.1%); *p* = 0.148] between intervention and comparison sites, reaching a high of 15.8% in the baseline period of the comparison sites (Table 1, Figure 2).

Using exit surveys, we observed that a total of two women initiated PrEP during the study period and a total of 30 women continued PrEP from prior initiations during the study period, limiting statistical comparison of PrEP initiation and continuation. Using record abstraction, we observed a total of 189 people who initiated PrEP during the study period and 357 who continued PrEP from prior initiations during the study period; it was not possible to distinguish women who initiated PrEP at MCH from all other people initiating PrEP recruited from other clinics within the facility. Overall, PrEP initiations increased in the comparison facilities more than in the intervention sites (comparison: 39–51; intervention: 43–56), while PrEP refills increased in the intervention facilities but decreased in the comparison sites (comparison: 127 –69; intervention: 79–82).

HCW perceptions of acceptability and appropriateness of the implementation strategy bundle were high; out of 20 possible points, acceptability scores had a median of 20.0 (IQR: 16.0, 20.0) and appropriateness scores had a median of 19.5 (IQR: 16.0, 20.0) (Table 2).

**Table 2:** Satisfaction, acceptability, appropriateness.

	Comparison sites		Intervention sites	
	Pre (*N* = 480)	Post (*N* = 478)	Pre (*N* = 480)	Post (*N* = 481)
	Mean (SD)	Mean (SD)	Mean (SD)	Mean (SD)
Client satisfaction				
Quality of the service^a^	3.04 (0.60)	3.26 (0.62)	3.14 (0.62)	3.26 (0.59)
Received the kind of service the client wanted^b^	3.61 (0.68)	3.69 (0.52)	3.48 (0.62)	3.69 (0.54)
The extent to which this facility met your needs^c^	3.53 (0.70)	3.58 (0.55)	3.36 (0.64)	3.54 (0.56)
Would recommend this facility to a friend^b^	3.84 (0.45)	3.89 (0.34)	3.70 (0.55)	3.91 (0.28)
Satisfied with the amount of help received^d^	3.50 (0.73)	3.51 (0.68)	3.29 (0.70)	3.50 (0.64)
Would come back to the facility^b^	3.85 (0.43)	3.89 (0.33)	3.72 (0.52)	3.92 (0.28)
Overall (out of 24 points)	21.36 (2.83)	21.81 (2.30)	20.70 (2.74)	21.82 (2.06)
HCW perceptions of appropriateness and acceptability of implementation strategy bundle				Post (*N* = 39)
				Mean (SD)
Appropriateness (IAM)^e^				
Fitting	–	–	–	4.55 (0.64)
Suitable	–	–	–	4.60 (0.55)
Applicable	–	–	–	4.60 (0.55)
A good match	–	–	–	4.55 (0.55)
Acceptability (AIM)^f^				
Meets approval	–	–	–	4.55 (0.64)
Appealing	–	–	–	4.56 (0.55)
I like it	–	–	–	4.50 (0.60)
I welcome it	–	–	–	4.58 (0.55)

^a^ Likert scale options: poor to excellent: 1–4.

^b^ Likert scale options: no, definitely not to yes, definitely: 1–4.

^c^ Likert scale options: none of my needs have been met to almost all of my needs have been met: 1–4.

^d^ Likert scale options: not satisfied to very satisfied: 1–4.

^e^ Average on 4-item Intervention Appropriateness Measure (IAM) scale; Likert scale (disagree to agree: 1–5).

^f^ Average on 4-item Acceptability of Intervention Measures (AIM) scale; Likert scale (disagree to agree: 1–5).

### PrEP knowledge score component changes

Client knowledge questions included items related to PrEP for HIV prevention, frequency of PrEP taking, time to reach maximum protection, concurrent condom use, PrEP side effects, and when to discontinue PrEP. The implementation strategy bundle was associated with an increase in accurate answers regarding PrEP use for HIV prevention of 28.6% percentage points (95% CI: 20.3, 37.0%; *p* < 0.001), reaching a high of 84.8%. Clients' knowledge of the frequency of PrEP use increased by 44.3% percentage points (95% CI: 36.6, 52.0%; *p* < 0.001), reaching a high of 66.7%. Accurate answers on the time PrEP takes to reach maximum protection increased by 29.1% percentage points (95% CI: 23.6, 34.5%; *p* < 0.001), reaching a high of 38.3%. Clients' knowledge of the concurrent use of condoms while taking PrEP increased by 21.4% percentage points (95% CI: 13.3, 29.4%; *p* < 0.001), reaching a high of 44.7%. PrEP side effects knowledge increased by 23.7% percentage points (95% CI: 19.0, 28.4%; *p* < 0.001), reaching a high of 29.3%. Knowledge of when a client can discontinue PrEP increased by 25.2% percentage points (95% CI: 19.4, 31.0%; *p* < 0.001), reaching a high of 38.0% (Table 3, Figure 2).

**Table 3:** Prep knowledge.

	Comparison sites				Intervention sites				Difference in difference [Change (intervention)—Change (comparison)] ***Adjusted for first ANC		
	*n*	%	*n*	%	*n*	%	*n*	%			
Indicator	Pre	Comparison: Pre	Post	Comparison: Post	Pre	Intervention: Pre	Post	Intervention: Post	Point estimate	95% CI	*p*-value
HIV prevention	188	39.2%	239	50.0%	217	45.2%	408	84.8%	28.6%	20.3%, 37.0%	<0.001
Frequency of use	108	22.5%	122	25.5%	94	19.6%	321	66.7%	44.3%	36.6%, 52.0%	<0.001
Time to protection	12	2.5%	37	7.7%	20	4.2%	184	38.3%	29.1%	23.6%, 34.5%	<0.001
Condom use	133	27.7%	138	28.9%	107	22.3%	215	44.7%	21.4%	13.3%, 29.4%	<0.001
Side effects	5	1.0%	16	3.3%	16	3.3%	141	29.3%	23.7%	19.0%, 28.4%	<0.001
Discontinuation	22	4.6%	38	7.9%	46	9.6%	183	38.0%	25.2%	19.4%, 31.0%	<0.001

### Hypothetical best possible performance with optimization

We calculated the hypothetical expected number of women who might be offered PrEP and accept PrEP if PrEP counseling, PrEP risk assessment, HIV testing, and offer were optimized, without changes to the proportion accepting PrEP. Among the 1,919 women who accessed care, if 77% had any risk indication for PrEP, 41% were eligible for HIV testing that day, and 99% of those tested were HIV negative—as observed within this dataset—a total of 588 women would have been offered PrEP. If 3.9% of those offered PrEP (2 initiations / 51 PrEP offers observed in this study) initiated PrEP, a total of 23 women would have initiated PrEP, approximately 12 times as many as were observed to have initiated PrEP in this study.

## Discussion

In this study, we observed that an implementation strategy package with video information, HIVST, and co-location of medication dispensing enhanced PrEP delivery in terms of implementation and service outcomes and client satisfaction, while not meaningfully increasing wait time or decreasing provider-client contact time. There were significant improvements in PrEP penetration, client satisfaction, and client PrEP knowledge. There was a significant but small increase in waiting time and no change in service delivery time. There was a trend for substantial improvement in PrEP fidelity. The strategy was associated with more pronounced effects in fidelity, penetration, and PrEP offer among clients seeking first ANC services compared to other visit types.

While implementation science focused on PrEP delivery has expanded in the past several years, more of this work focuses on other priority populations than pregnant and postpartum women (34–39). Unlike some other priority populations, pregnant and postpartum women are already presenting to a status-neutral MCH care clinic where they receive sequenced integrated care for a range of health conditions. Attendance at ANC clinics is remarkably high in Kenya and many sub-Saharan African countries (40). The last step of the PrEP cascade of PrEP acceptance can be addressed through demand creation. However, the earlier steps of the PrEP cascade—PrEP counseling, risk screening, information provision, HIV testing, and PrEP offer—are well-suited to supply-side strategies. The aforementioned recent systematic review of implementation science focused on pregnant and postpartum women noted that most implementation strategies tested for this population intervened at the intrapersonal and interpersonal levels, rather than at the systems level, as the present study does. It called for testing supply-side strategies in order to improve implementation and realize the population-level benefits of PrEP for HIV prevention for pregnant women (10). We note that video education can serve both as a supply-side strategy—by shifting standard information provision from healthcare workers to automated provision—as well as a demand-generating strategy.

We observed greater improvements in knowledge scores associated with the implementation strategy package that contained video education. Video education has been tested in resource-rich and resource-limited settings; as mentioned above, a recent systematic review found that video education was as effective or more effective than counselor-delivered information (29). Standard videos are well-suited to resource-limited contexts by overcoming a variety of structural barriers; video education allows limited HCW time to be focused on individualized post-test counseling, provides standard information that can be rapidly updated faster than large cadres can receive refresher training, can present information in multiple languages, and can be delivered in group settings. In a Kenyan study testing video education for HIV testing, video education—both in an individual and group format—was associated with higher knowledge scores than counselor-delivered sessions (30). It is possible that video education provided in the waiting room in the present study allowed HCWs to provide more PrEP-related services—such as risk screening and counseling—in a fixed visit time. Waiting and service time were not substantially different between intervention and comparison clinics.

HIVST for PrEP initiation has not been widely tested, despite the widespread use of HIVST in non-facility settings and some use of HIVST at MCH clinics. A recent systematic review of HIVST for PrEP initiation and continuation found limited trial data supporting HIVST for PrEP continuation and no comparative study results testing HIVST for PrEP initiation but noted several ongoing studies (41). This presents the first data demonstrating the use of HIVST for PrEP initiation in a real-world setting. In our study, HIVST was offered only to women who had received standard HIV testing at a prior ANC visit; while this added complexity, it was deemed necessary for women to have had prior counseling experience in standard HIV testing. A prior study conducted by Oyaro et al. in Kenya also utilized HIVST for repeat maternal HIV testing in MCH clinics; just over half of women elected HIVST instead of counselor-delivered blood-based testing, citing privacy, ease, and speed as major factors (31). Substantial logistical coordination was necessary to facilitate the use of HIVST in busy waiting rooms, ensuring confidentiality and proper test performance, as well as confirmatory reading by HCWs; privacy booths were utilized following the experience described by Oyaro et al.

Co-locating PrEP dispensing services in the MCH clinic rather than at a central facility pharmacy or an HIV care-specific pharmacy was tested to improve flow, efficiency, and acceptability for women. Prior qualitative studies in Kenya with pregnant and postpartum women highlighted that women not living with HIV did not want to receive their HIV prevention medications from an HIV care-specific pharmacy for reasons of stigma (17). Prior quantitative studies noted that integrated delivery in MCH was feasible in models with additional healthcare workers (16, 24). A study in Kenya that tested a “one-stop-shop” model—including co-location of dispensing and services, provider cross-training and task shifting, and shifting to a lower volume clinic—observed a decrease in waiting time, no change in PrEP initiations and continuation, and high acceptability of the model due to decreased stigma and increased privacy (42). In our study, we observed a small increase in waiting time, unlike the one-stop-shop model, but similarly observed higher client satisfaction and no change in provider service time.

Within this study, we were able to use exit surveys to readily measure and compare implementation outcomes but were not able to meaningfully compare clinical outcomes—such as PrEP initiation and continuation changes—due to low frequency. Conversely, we were able to use routine records to compare PrEP initiations and continuation events, but not PrEP implementation outcomes due to limited fields collected in register data. However, PrEP initiation and continuation records do not specify the location from which a client was referred or initiated, limiting the use of this data source to assess the impact of implementation strategy testing within certain clinics within a site. While revisions to PrEP and MCH registers are expected in the near future in Kenya, it will likely remain necessary to include both primary data and record abstraction to meaningfully assess the impact of implementation strategies across the PrEP cascade.

While we noted substantial improvements in implementation outcomes associated with this implementation strategy package, large gaps remained in absolute coverage for each step. Two steps that could be optimized simply, namely, PrEP penetration (being talked to about PrEP today) and PrEP offer among eligible women, remained below 10% and 16%, respectively, even in intervention clinics. Additional implementation strategies that prompt providers to offer consistent services to each client—such as checklists, inclusion in standard registers, and other “nudge” strategies—should be tested in the future to close these noted gaps in implementation and offer high-quality and consistent services to clients. Improvements were more pronounced among women seeking their first ANC visit; this visit may offer an additional opportunity to nudge for high coverage of PrEP penetration and offer. Additionally, levels of uptake of PrEP in pregnant and postpartum populations are widely variable, with staffing being a likely determinant of uptake. The PrIYA and PrIMA studies in Kenya both offered integrated PrEP in MCH in western Kenya and included additional research staff that delivered services; uptake of PrEP was 22% and 18.6% in PrIYA and PrIMA, respectively (16, 24). In contrast, the same sites that participated in the PrIYA study were assessed after study staff departed; uptake was substantially lower at 4% (22), which was similar to the 3.9% uptake noted in the present study. Approaches are needed to address the large differences in uptake of PrEP that appear to be partially related to staffing, especially as the field looks forward to national scale-up.

Our study has several limitations. While we aimed to test implementation strategies in real-world contexts, without additional research staff for delivery, certain resources, such as purchasing privacy booths and televisions, were necessary in order to activate the strategy. These were purchased using research rather than program funds, limiting external validity. Additionally, there were external factors that influenced service provision, including three HCW strikes spread over 5 weeks; the frequency and duration of these strikes were balanced between the intervention and comparison clinics and nearly balanced between the pre-intervention and post-intervention periods, having minimal impact on our difference in difference analysis approach. Were these interruptions not present, we would have expected a larger magnitude difference associated with the implementation strategy bundle. While we initially aimed to conduct a controlled interrupted time series analysis, the interruptions in data collection necessitated a switch to a difference-in-differences analytic approach, which has poorer control for baseline temporal trends. We assessed PrEP penetration by asking if “someone ‘talked’ to a client about PrEP today”; while this was intended to assess the number of women receiving basic information about PrEP by a counselor or by video, women who received information by video likely answered “no” more frequently. This assumption is further amplified by large observed improvements in knowledge among women in the intervention groups. We may have systematically underestimated PrEP penetration in the intervention group during the post-intervention period, which would have underestimated the magnitude of the association between the implementation strategy package and PrEP penetration. Future studies that assess PrEP penetration should assess learning information about PrEP through any facility-based interaction or experience. We were not able to collect process data on the number of women offered and accepting HIVST, the denominator of women eligible for HTS by facility-specific guidelines, nor information on whether their visits were specifically shorter, limiting our ability to further investigate the limited impact of this strategy on implementation. We allocated facilities to intervention vs. comparison conditions prior to initiating baseline data collection; therefore, we missed the opportunity to balance the distribution of PrEP outcomes between conditions, potentially contributing to a less robust parallel trends assumption for a difference in differences design. Finally, the facilities selected for this study are somewhat generalizable to the larger volume of health facilities in Siaya, Homa Bay, and Kisumu counties, but there may be a bias towards better-resourced facilities in terms of physical space.

## Conclusions

An implementation strategy package with video information, HIV self-testing, and co-location of medication dispensing enhanced integrated PrEP delivery for pregnant women in MCH clinics. There were significant improvements in PrEP penetration, client satisfaction, and client PrEP knowledge. The implementation strategy package was not associated with meaningfully increased wait time or decreased provider-client contact time. This package of strategies, which did not include additional healthcare workers or research staff, merits broader implementation, alongside additional strategies to close gaps in absolute coverage.

## Data Availability

The raw data supporting the conclusions of this article will be made available by the authors, without undue reservation.
