# Supplementary material for: An implementation strategy package (video education, HIV self-testing, and co-location) improves PrEP implementation for pregnant women in antenatal care clinics in western Kenya
Source: Front Reprod Health. 2023 Nov 17;5:1205503. doi: 10.3389/frph.2023.1205503 (PMC10690761; doi:10.3389/frph.2023.1205503)
Supplement: Supplementary file 1 [file Table3.docx]

**Supplementary Tables**

**Supplementary Table 1:** Difference in differences comparison of implementation, effectiveness, and service outcomes stratified by visit type (first ANC clients vs any other visit type)

|  | **Comparison sites** | | | | **Intervention sites** | | | | **Difference in difference** [(Change in intervention sites) – (Change in comparison sites)] **among first ANC clients** | | |  | **Comparison sites** | | | | **Intervention sites** | | | | **Difference in difference** [(Change in intervention sites) – (Change in comparison sites)] **among other visit type clients** | | |
| --- | --- | --- | --- | --- | --- | --- | --- | --- | --- | --- | --- | --- | --- | --- | --- | --- | --- | --- | --- | --- | --- | --- | --- |
|  | Pre (N=91) | | Post (N=89) | | Pre (N=129) | | Post (N=103) | | Point estimate | Confidence interval | p-value |  | Pre (N=389) | | Post (N=389) | | Pre (N=351) | | Post (N=378) | | Point estimate | Confidence interval | p-value |
| ***Outcome*** | n (%) or median IQR | N | n (%) or median IQR | N | n (%) or median IQR | N | n (%) or median IQR | N |  |  |  |  | n (%) or median IQR | N | n (%) or median IQR | N | n (%) or median IQR | N | n (%) or median IQR | N |  |  |  |
| **PrEP fidelity^1^** | 3 (5.0%) | 60 | 2 (2.7%) | 75 | 8 (9.2%) | 87 | 16 (17.6%) | 91 | 12.5% | (-0.3%, 25.2%) | 0.055 |  | 1 (1%) | 101 | 3 (3.4%) | 89 | 6 (7.4%) | 81 | 12 (13.3%) | 90 | 3.5% | (-6.2%, 13.2%) | 0.474 |
| HIV testing^3^ | 55 (91.7%) | 60 | 72 (96%) | 75 | 83 (95.4%) | 87 | 89 (97.8%) | 91 | -1.9% | (-11.2%, 7.3%) | 0.661 |  | 43 (41.8%) | 103 | 35 (39.3%) | 89 | 59 (72.0%) | 82 | 55 (61.1%) | 90 | -1.8% | (-20.6%, 17.0%) | 0.851 |
| PrEP risk screening^3^ | 16 (18%) | 91 | 49 (55%) | 89 | 53 (41.1%) | 129 | 73 (70.9%) | 103 | -9.4% | (-26.9%, 8.0%) | 0.288 |  | 27 (6.9%) | 389 | 55 (14.1%) | 389 | 93 (26.5%) | 351 | 86 (22.8%) | 378 | -10.8% | (-18.2%, 3.3%) | 0.004 |
| **PrEP penetration^1^** | 3 (3%) | 91 | 3 (3%) | 89 | 9 (7.0%) | 129 | 19 (18.4%) | 103 | 12.7% | (2.2%, 23.1%) | 0.017 |  | 14 (3.6%) | 388 | 5 (1.3%) | 389 | 21 (6.0%) | 349 | 27 (7.1%) | 378 | 3.4% | (-0.7%, 7.6%) | 0.106 |
| PrEP offer^3^ | 3 (3%) | 91 | 5 (6%) | 89 | 2 (1.6%) | 129 | 12 (11.7%) | 103 | 7.9% | (-0.8%, 16.6%) | 0.074 |  | 5 (1.3%) | 389 | 4 (1.0%) | 386 | 4 (1.1%) | 348 | 16 (4.2%) | 377 | 3.3% | (0.5%, 6.1%) | 0.019 |
|  | 2 (15.4%) | 13 | 3 (7.0%) | 43 | 2 (4.4%) | 46 | 7 (10.9%) | 64 | 15.2% | (-4.9%, 35.3%) | 0.139 |  | 1 (16.7%) | 6 | 3 (16.7%) | 18 | 2 (6.3%) | 32 | 6 (20.7%) | 29 | 14.4% | (-21.6%, 50.5%) | 0.432 |
| PrEP uptake^2^ | 0 (0%) | 3 | 0 (0%) | 5 | 0 (0%) | 2 | 0 (0%) | 12 | -- | -- | -- |  | 1 (20%) | 5 | 1 (25%) | 4 | 0 (0%) | 4 | 0 (0%) | 16 | -- | -- | -- |
|  | 0 (0%) | 91 | 0 (0%) | 89 | 0 (0%) | 129 | 0 (0%) | 103 | -- | -- | -- |  | 1 (0.3%) | 389 | 1 (0.3%) | 389 | 0 (0%) | 351 | 0 (0%) | 378 | -- | -- | -- |
| PrEP continuation^2^ | 1 (33.3%) | 3 | 2 (40.0%) | 5 | 0 (0%) | 2 | 0 (0%) | 12 | -- | -- | -- |  | 0 (0%) | 5 | 0 (0%) | 4 | 1 (25%) | 4 | 1 (6.3%) | 16 | -- | -- | -- |
|  | 2 (2.2%) | 91 | 5 (5.6%) | 89 | 2 (1.6%) | 129 | 0 (0%) | 103 | -- | -- | -- |  | 3 (0.8%) | 389 | 5 (1.3%) | 389 | 7 (2.0%) | 351 | 6 (1.6%) | 378 | -- | -- | -- |
| **Client satisfaction^1^** | 22.0 (20.0, 23.0) | 91 | 22.0 (20.0, 23.0) | 89 | 21.0 (19.0, 23.0) | 129 | 22.0 (20.0, 23.0) | 103 | 0.74 | (-0.20, 1.68) | 0.122 |  | 23.0 (20.0, 23.0) | 389 | 23.0 (21.0, 24.0) | 389 | 21.0 (19.0, 23.0) | 351 | 23.0 (21.0, 23.0) | 378 | 0.63 | (0.14, 1.13) | 0.012 |
| Client PrEP knowledge^2^ | 0 (0%) | 91 | 1 (1.1%) | 89 | 2(1.6%) | 129 | 16 (15.5%) | 103 | 11.9% | (4.1%, 19.7%) | 0.003 |  | 0 (0%) | 389 | 4 (1.0%) | 389 | 3(1.0%) | 351 | 40 (10.6%) | 378 | 8.9% | (5.5%, 12.2%) | <0.001 |
|  | 0.868 | 91 | 1.393 | 89 | 1.093 | 129 | 3.223 | 103 | 1.6 | (1.00, 2.20) | <0.001 |  | 1 | 389 | 1.2 | 389 | 1.02 | 351 | 3 | 378 | 1.7 | (1.44, 2.04) | <0.001 |

**Supplementary Table 2:** Demographic characteristics

|  |  | Comparison sites | | Intervention sites | |
| --- | --- | --- | --- | --- | --- |
|  | Overall | Pre | Post | Pre | Post |
|  | n (%) or Median (IQR) | | | | |
| Age | 25.0 (22.0 - 30.0) | 25.0 (21.0 - 30.0) | 25.0 (22.0 - 30.0) | 24.0 (21.0 - 29.0) | 25.0 (22.0 - 30.0) |
| First ANC | 412 (21.5%) | 91 (19.0%) | 89 (18.6%) | 129 (26.9%) | 103 (21.4%) |

**Supplementary Table 3**: Site specific indicators by pre- post- period

| **Pre-period** | **Site 1** | **Site 2** | **Site 3** | **Site 4** | **Site 5** | **Site 6** | **Site 7** | **Site 8** | **Range: Pre period** |
| --- | --- | --- | --- | --- | --- | --- | --- | --- | --- |
| PrEP penetration | 1.7% | 6.7% | 2.5% | 4.2% | 5.8% | 10.0% | 0.0% | 8.3% | 0% - 10% |
| PrEP fidelity | 0.0% | 7.0% | 0.0% | 0.0% | 0.0% | 16.0% | 0.0% | 12.0% | 0% - 16% |
| PrEP offer | 2.5% | 4.2% | 0.0% | 0.0% | 0.0% | 2.5% | 0.0% | 2.5% | 0% - 4.2% |
| PrEP offer among eligible | 0.0% | 13.0% | 0.0% | 0.0% | 0.0% | 5.0% | 0.0% | 5.0% | 0% - 13% |
| PrEP knowledge | 0.0% | 0.0% | 0.0% | 0.8% | 0.0% | 1.7% | 0.0% | 1.7% | 0% - 1.7% |
| HIV testing | 95.0% | 42.0% | 85.0% | 71.0% | 68.0% | 86.0% | 58.0% | 88.0% | 42% - 95% |
| Client satisfaction | 23.0 (22.0 - 23.0) | 20.0 (18.0 - 22.0) | 23.0 (21.0 - 23.0) | 21.0 (19.0 - 22.0) | 23.0 (22.0 - 23.0) | 20.0 (18.0 - 22.0) | 23.0 (21.0 - 23.0) | 21.0 (19.0 - 23.0) | 21-23 |
| Time spent receiving services | 16.5 (10 - 36) | 17 (12 - 36) | 19 (10.5 - 30) | 25.5 (13.5 - 59) | 12 (6 - 33) | 17.5 (13.5 - 28.5) | 16 (11 - 25.5) | 17 (10 - 45) | 12-25.5 |
| Time spent waiting for services | 76 (49.5 - 111) | 41.5 (23 - 71.5) | 79 (41 - 124.5) | 44.5 (19.5 - 65) | 36 (15 - 57.5) | 50 (20 - 75.5) | 10.5 (5.5 - 25) | 37.5 (25 - 56.5) | 10.5-79 |
|  |  |  |  |  |  |  |  |  |  |
| **Post-period** | **Site 1** | **Site 2** | **Site 3** | **Site 4** | **Site 5** | **Site 6** | **Site 7** | **Site 8** | **Range: Post period** |
| PrEP penetration | 3.4% | 0.8% | 14.2% | 2.5% | 1.7% | 12.4% | 0.8% | 9.2% | 0.8% - 14.2% |
| PrEP fidelity | 6.0% | 2.0% | 24.0% | 5.0% | 3.0% | 21.0% | 0.0% | 13.0% | 0% - 24.0% |
| PrEP offer | 2.5% | 0.8% | 6.7% | 0.8% | 2.5% | 8.4% | 1.7% | 7.5% | 0.8% - 8.4% |
| PrEP offer among eligible | 8.0% | 4.0% | 26.0% | 0.0% | 9.0% | 16.0% | 9.0% | 12.0% | 0.0% - 26.0% |
| PrEP knowledge | 0.0% | 4.2% | 8.3% | 10.8% | 0.0% | 5.8% | 0.0% | 21.7% | 0.0% - 21.7% |
| HIV testing | 81.0% | 61.0% | 66.0% | 72.0% | 61.0% | 88.0% | 55.0% | 89.0% | 55.0% - 89.0% |
| Client satisfaction | 23.0 (20.0, 24.0) | 21.5 (20.0, 23.0) | 23.0 (21.0, 23.0) | 22.0 (20.0, 23.0) | 23.0 (21.0, 24.0) | 23.0 (21.0, 23.0) | 23.0 (21.0, 23.0) | 22.0 (20.0, 23.0) | 21.5 - 23.0 |
| Time spent receiving services | 15 (12, 24.5) | 12 (7.5, 32) | 17 (10, 27) | 16.5 (10.5, 37.5) | 12 (7.5, 31.5) | 17.5 (12.5, 27) | 15 (11, 30.5) | 15 (9.5, 40.5) | 12.0 - 17.5 |
| Time spent waiting for services | 43 (31, 80.5) | 32 (17.5, 51.5) | 72 (40.5, 101) | 49.5 (36.5, 70) | 29 (17, 40.5) | 63.5 (37, 89) | 14 (7.5, 21.5) | 37.5 (17, 63.5) | 14.0-72.0 |

**Supplementary Table 4:** Site specific details

| **Facility** | **Level** | **County** | **Volume (High vs Low)*** |
| --- | --- | --- | --- |
| Site 1 | Sub county | Siaya County | High Volume |
| Site 2 | Health center | Siaya County | Low Volume |
| Site 3 | County referral | Siaya County | High Volume |
| Site 4 | Sub county | Siaya County | High Volume |
| Site 5** | Sub county | Kisumu County | High Volume |
| Site 6** | Sub county | Kisumu County | High Volume |
| Site 7 | Health center | Homabay county | Low Volume |
| Site 8 | Sub county | Homabay county | High Volume |

**We used a cut off of 3000 antenatal clients per month to distinguish between high and low volume sites*

***Site previously engaged in a research trial*
